# Supplementary material for: Graphene Nanoplatelets-Based Textured Polymeric Fibrous Fabrics for the Next-Generation Devices
Source: Polymers (Basel). 2022 Dec 10;14(24):5415. doi: 10.3390/polym14245415 (PMC9785025; doi:10.3390/polym14245415)
Supplement: Supplementary file 1 [file polymers-14-05415-s001.zip › polymers-2067654-supplementary.pdf]

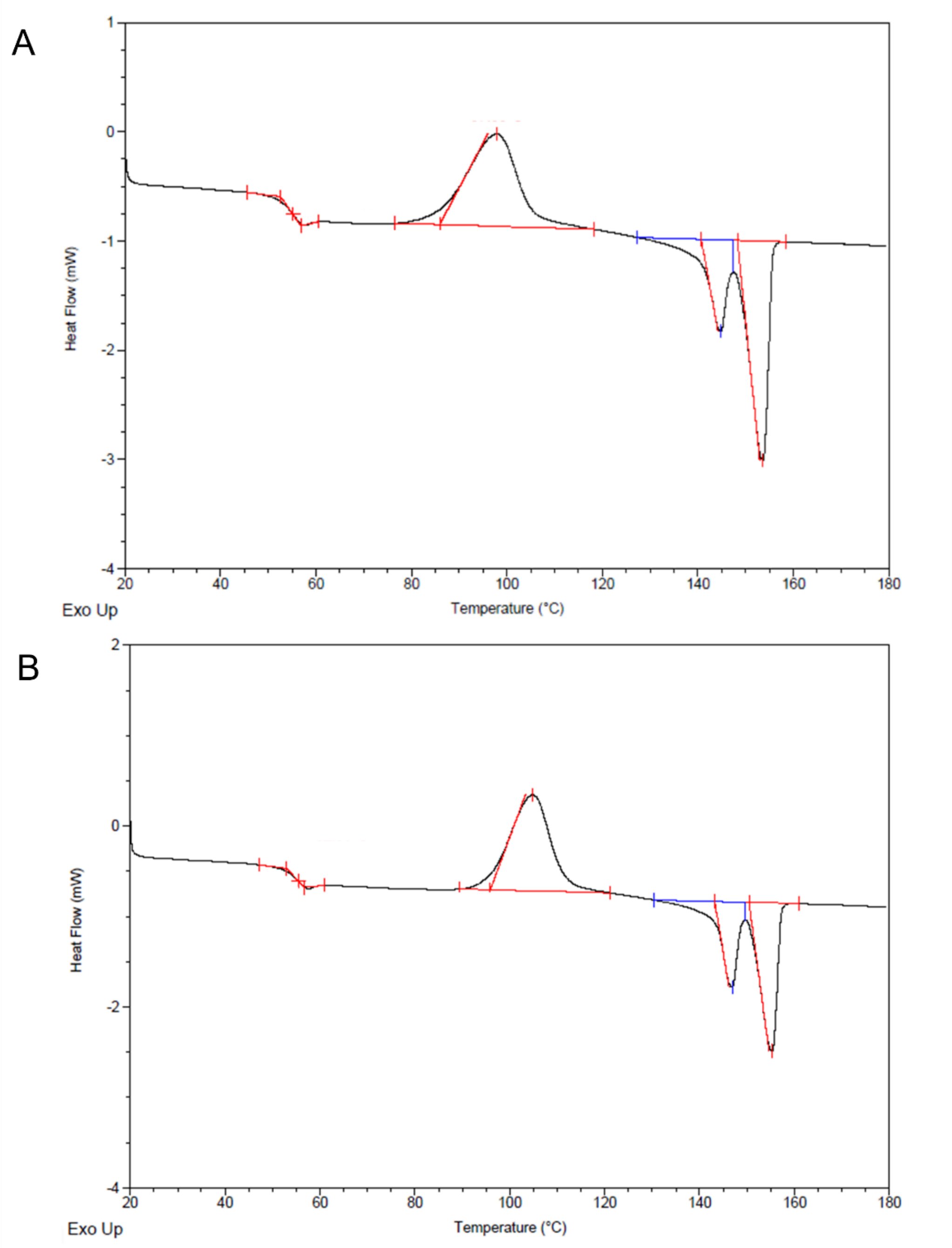

Figure S1. Representative second heating thermographs for PLA-nCD (A) and PLA (B) at the heating rate of 2 K/min.

A

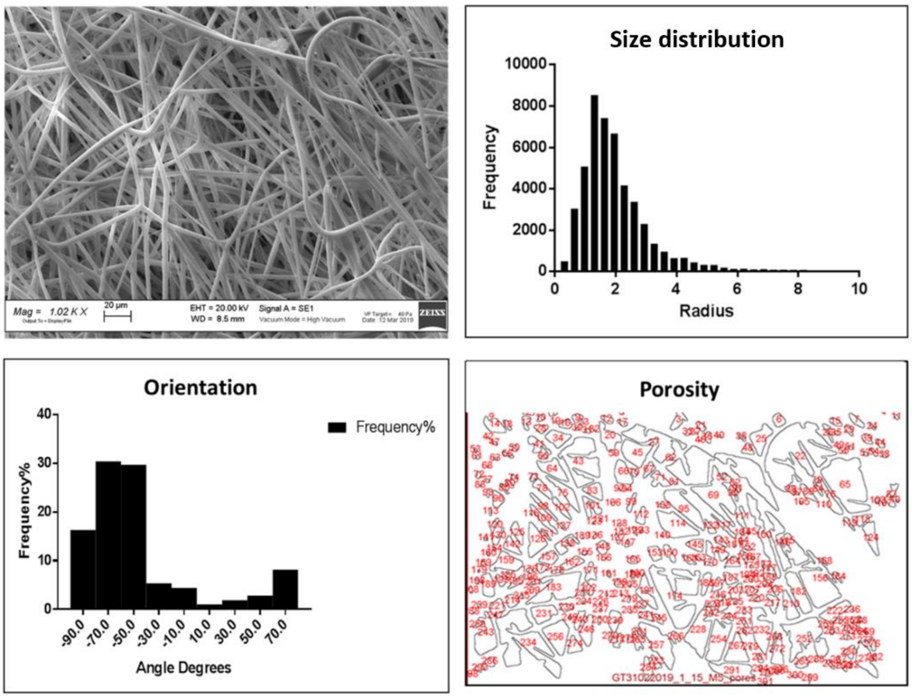

Mag = 1.02 K X
20 µm
EHT = 20.00 kV
WD = 8.5 mm
Signal A = SE1
Vacuum Mode = High Vacuum
ZEISS
Size distribution
Frequency
Radius
Orientation
Frequency%
Angle Degrees
Porosity

B

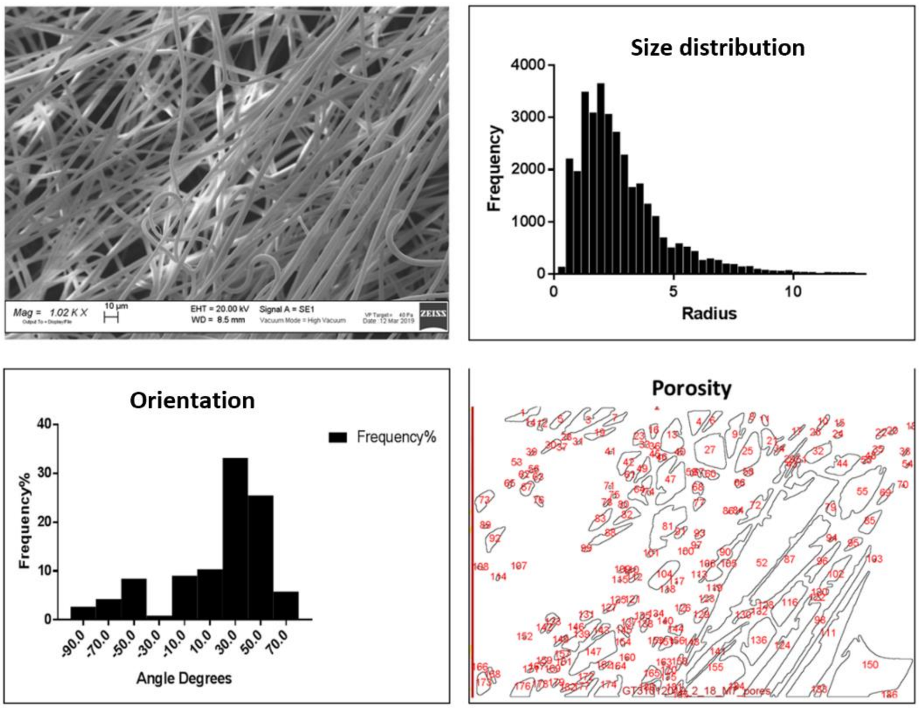

Mag = 1.02 K X
10 µm
EHT = 20.00 kV
WD = 8.5 mm
Signal A = SE1
Vacuum Mode = High Vacuum
ZEISS
Size distribution
Frequency
Radius
Orientation
Frequency%
Angle Degrees
Porosity

C

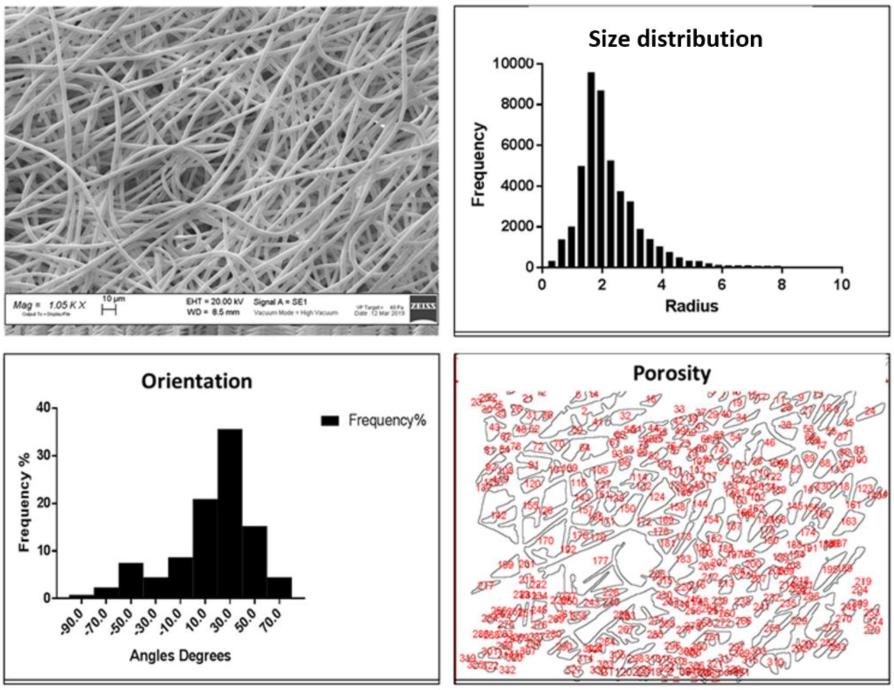

D

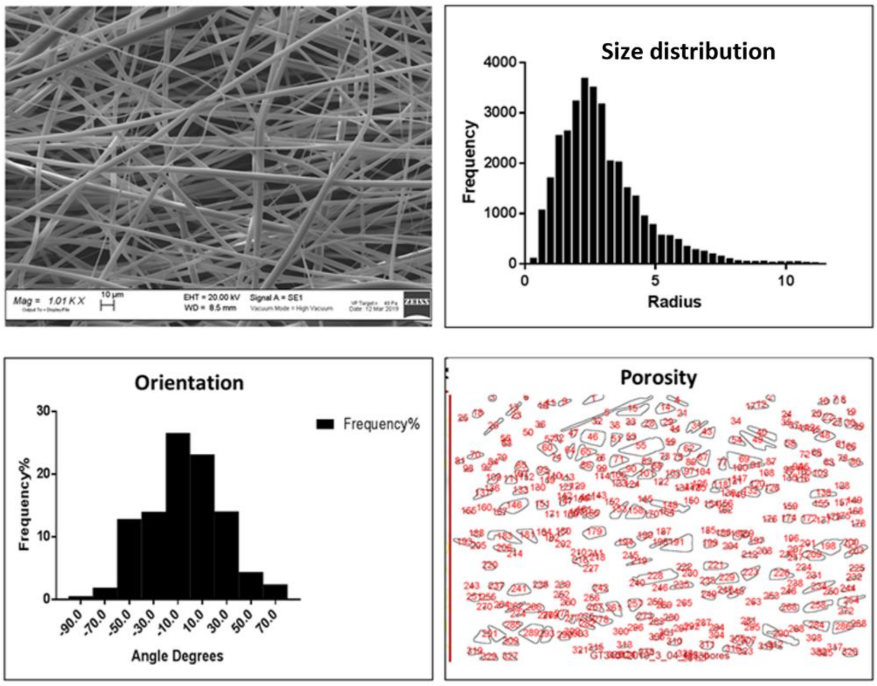

Figure S2. Morphological examination of PLA matrices obtained by using MC as solvent. SEM images of electrospun matrices (1 KX of magnification, scale bar=10 μm): **(A) F-PLA,** (**B) R-PLA,** (**C) S-PLA,** (**D) D-PLA**.

Images software elaboration leads to the evaluation of fiber size distribution, fiber orientation frequency and mat porosity.

A

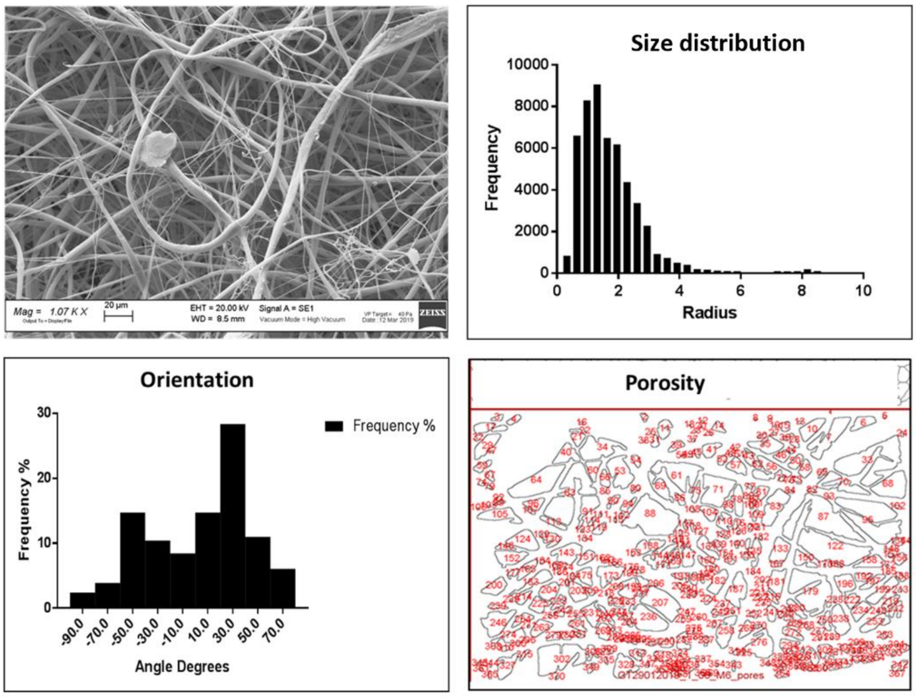
Mag = 1.07 K X
20 µm
EHT = 20.00 kV
WD = 8.5 mm
Signal A = SE1
Vacuum Mode = High Vacuum
Date :12 Mar 2019
ZEISS
Size distribution
Frequency
Radius
Orientation
Frequency %
Angle Degrees
Porosity

B

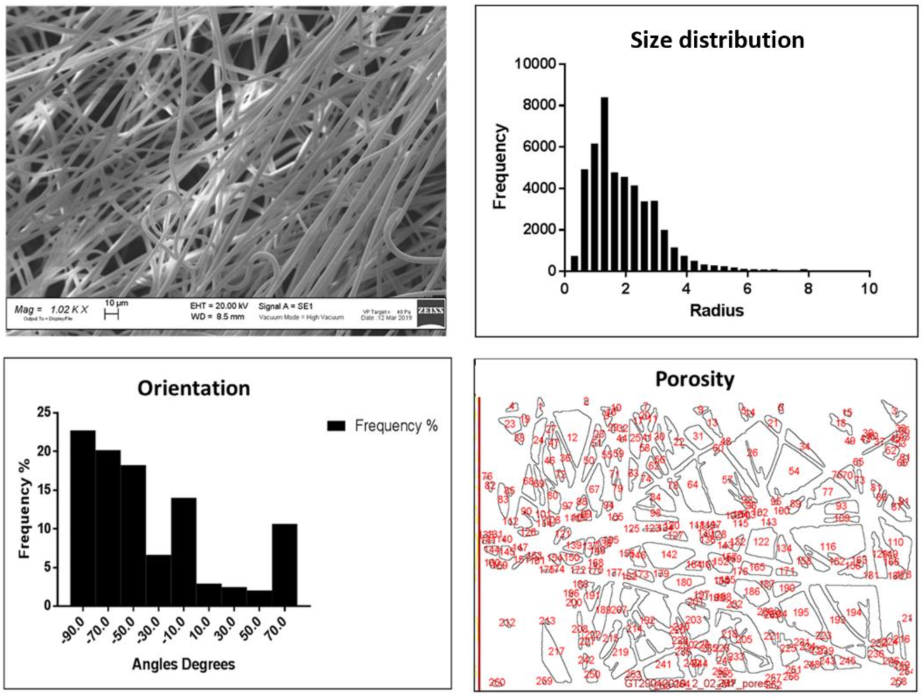
Mag = 1.02 K X
10 µm
EHT = 20.00 kV
WD = 8.5 mm
Signal A = SE1
Vacuum Mode = High Vacuum
Date :12 Mar 2019
ZEISS
Size distribution
Frequency
Radius
Orientation
Frequency %
Angles Degrees
Porosity

C

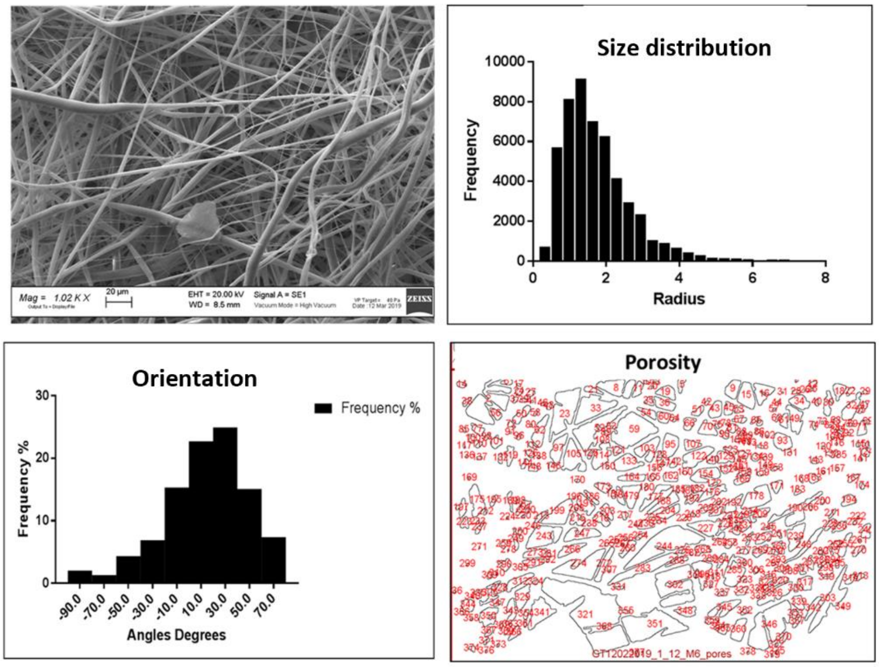

D

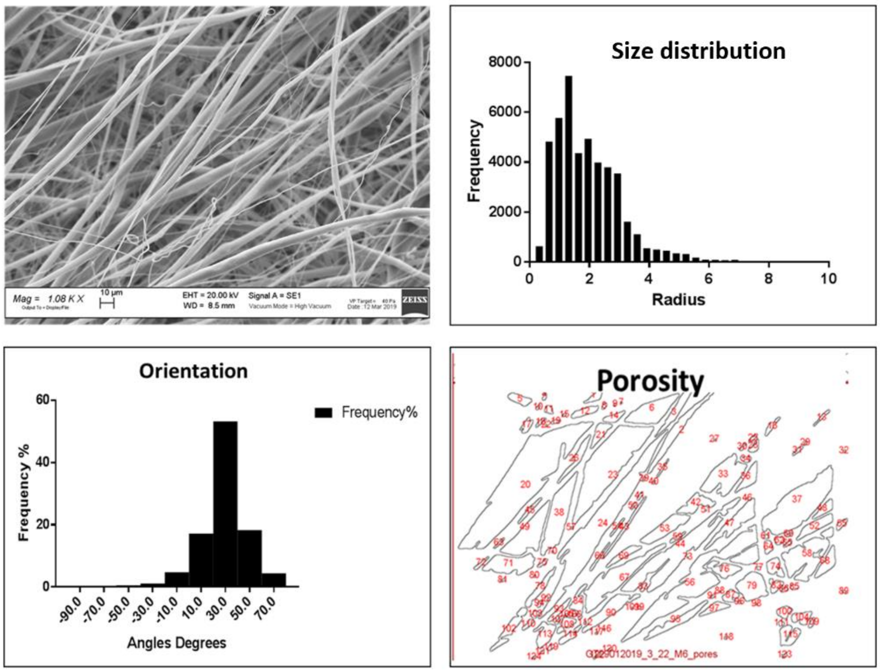

Figure S3. Morphological examination of PLA-nCD matrices obtained by using MC as solvent. SEM images of electrospun matrices (1 KX of magnification, scale bar=10 μm):**(A) F-PLA-nCD,** (**B) R-PLA-nCD,** (**C) S-PLA-,** (**D) D-PLA**. Images software elaboration leads to the evaluation of fiber size distribution, fiber orientation frequency and mat porosity.

A

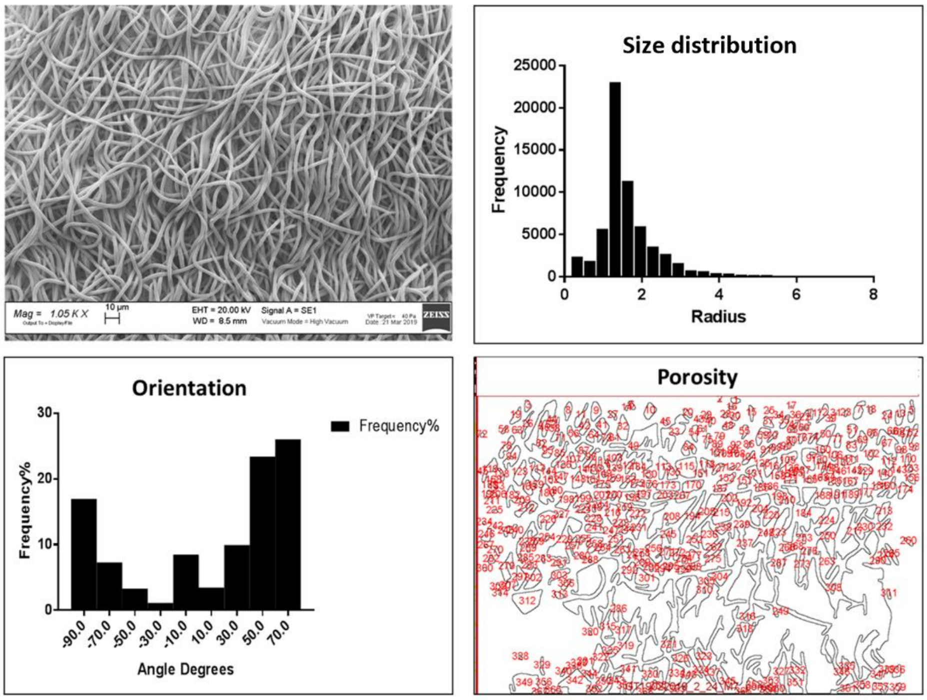

Mag = 1.05 K X
10 µm
EHT = 20.00 kV
WD = 8.5 mm
Signal A = SE1
Vacuum Mode = High Vacuum
Date :21 Mar 2019
Size distribution
Frequency
25000
20000
15000
10000
5000
0
0
2
4
6
8
Radius
Orientation
Frequency%
30
20
10
0
-90.0
-70.0
-50.0
-30.0
-10.0
10.0
30.0
50.0
70.0
Angle Degrees
Porosity

B

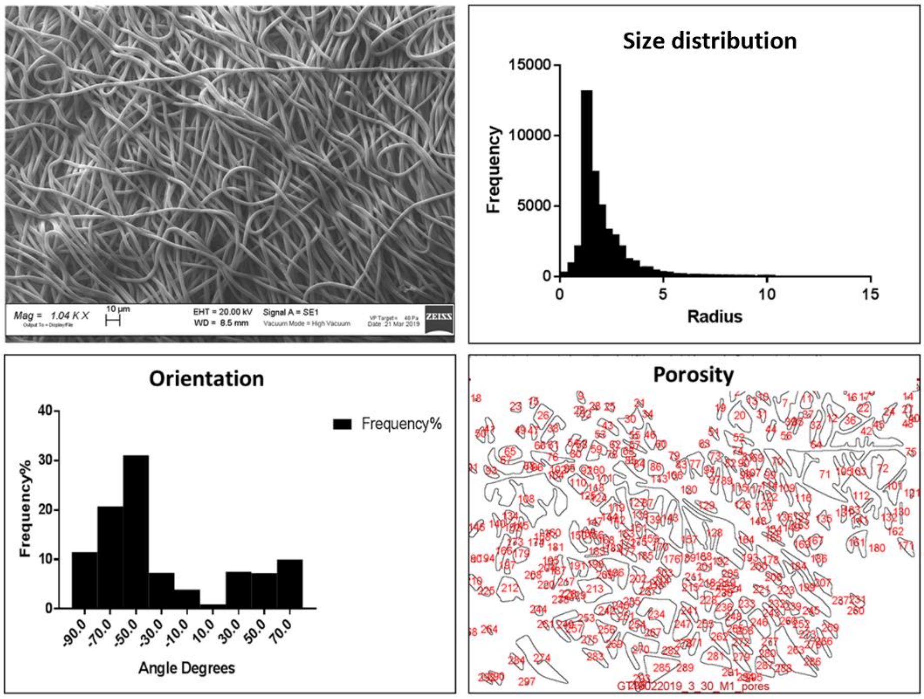

Mag = 1.04 K X
10 µm
EHT = 20.00 kV
WD = 8.5 mm
Signal A = SE1
Vacuum Mode = High Vacuum
Date :21 Mar 2019
Size distribution
Frequency
15000
10000
5000
0
0
5
10
15
Radius
Orientation
Frequency%
40
30
20
10
0
-90.0
-70.0
-50.0
-30.0
-10.0
10.0
30.0
50.0
70.0
Angle Degrees
Porosity

C

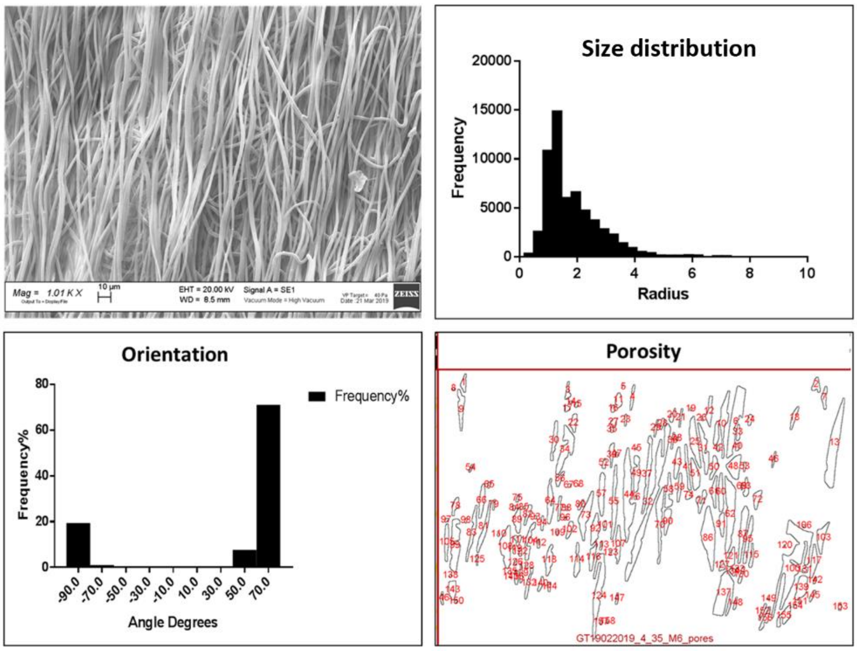

D

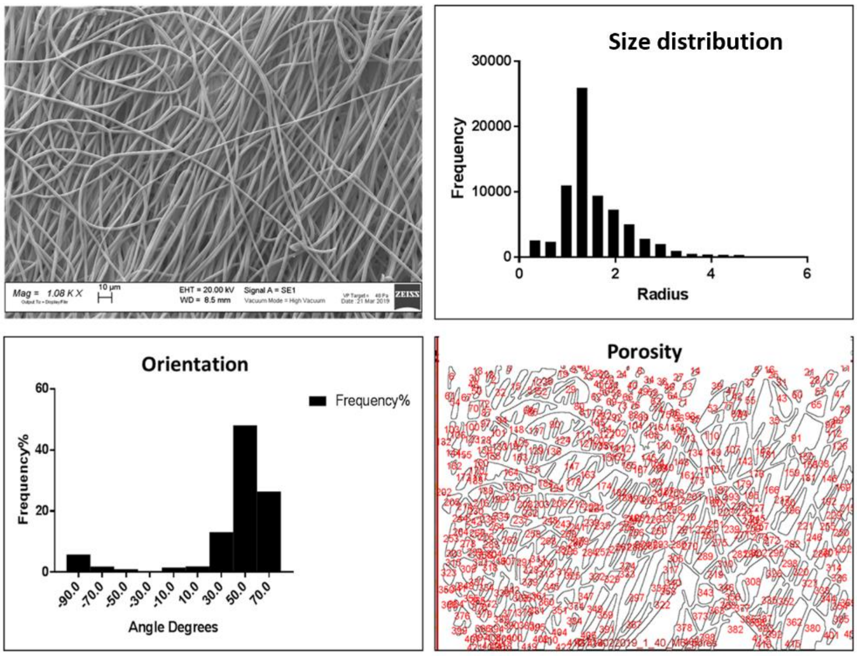

Figure S4. Morphological examination of PLA matrices obtained by using MC:DMF as solvent. SEM images of electrospun matrices (1 KX of magnification, scale bar=10 μm): **(A) F-PLA,** (**B) R-PLA,** (**C) S-PLA,** (**D) D-PLA**.

Images software elaboration leads to the evaluation of fiber size distribution, fiber orientation frequency and mat porosity.

A

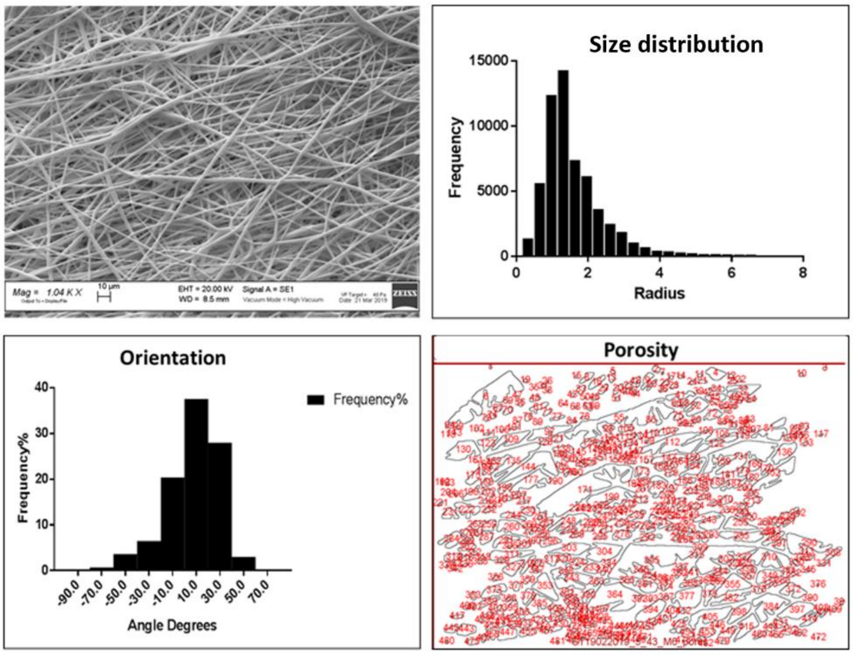
Mag = 1.04 K X
10 µm
EHT = 20.00 kV
WD = 8.5 mm
Signal A = SE1
Vacuum Mode = High Vacuum
Date: 21 Mar 2019
ZEISS
Size distribution
Frequency
Radius
Orientation
Frequency%
Angle Degrees
Porosity

B

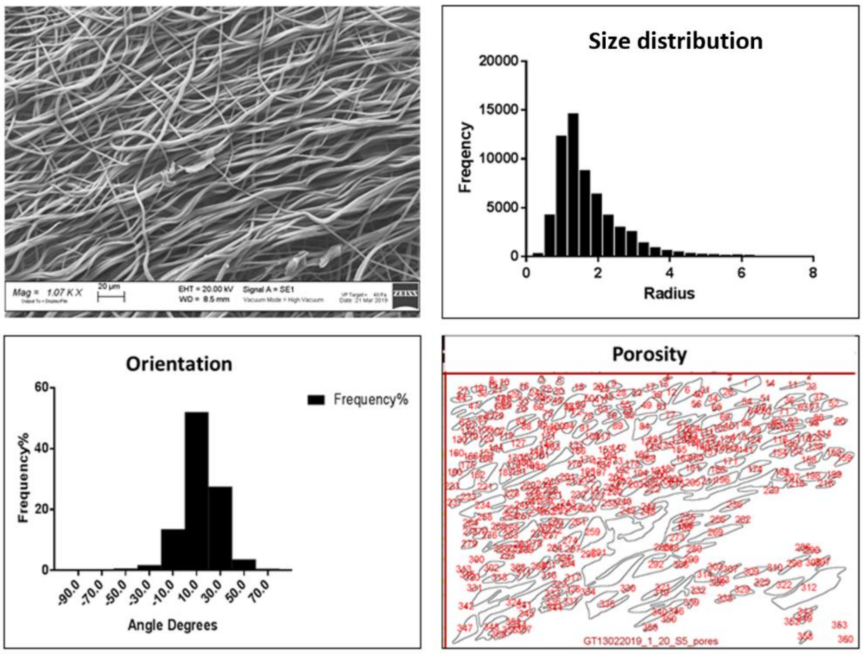
Mag = 1.07 K X
20 µm
EHT = 20.00 kV
WD = 8.5 mm
Signal A = SE1
Vacuum Mode = High Vacuum
Date: 21 Mar 2019
ZEISS
Size distribution
Freqency
Radius
Orientation
Frequency%
Angle Degrees
Porosity
GT13022019_1_20_S5_pores

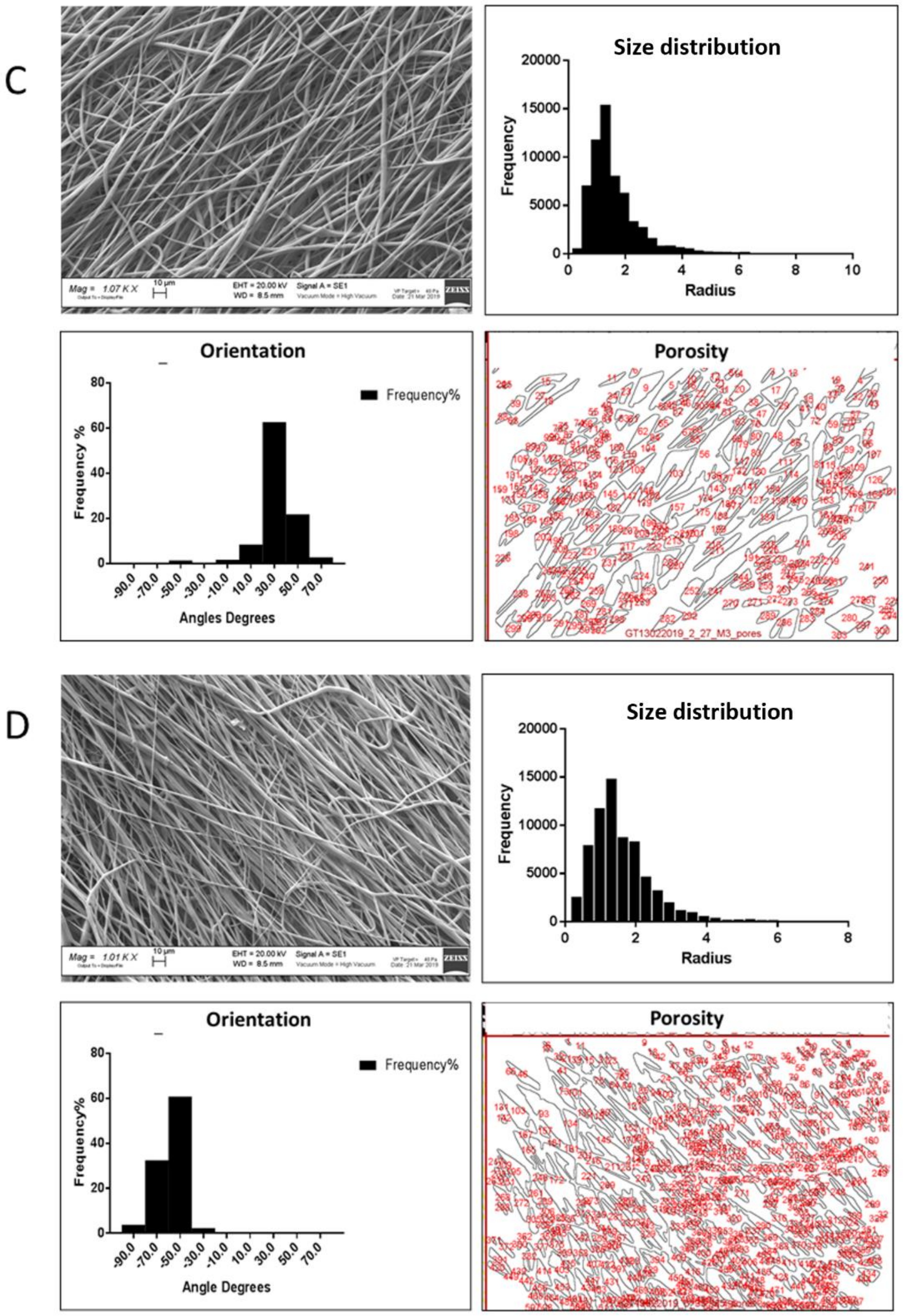

Figure S5. Morphological examination of PLA-nCD matrices obtained by using MC:DMF as solvent. SEM images of electrospun matrices (1 KX of magnification, scale bar=10 μm): **(A) F-PLA-nCD, (B) R-PLA-nCD, (C) S-PLA-nCD, (D) D-PLA-nC**.

Images software elaboration leads to the evaluation of fiber size distribution, fiber orientation frequency and mat porosity.
